# Phase II trial of the antiangiogenic agent IM862 in metastatic renal cell carcinoma

**DOI:** 10.1038/sj.bjc.6602126

**Published:** 2004-10-19

**Authors:** G Deplanque, S Madhusudan, P H Jones, S Wellmann, K Christodoulos, D C Talbot, T S Ganesan, A Blann, A L Harris

**Affiliations:** 1 University of Oxford, Cancer Research UK Medical Oncology Unit, The Churchill, Old Road, Oxford OX3 7LJ, England

**Keywords:** renal cell cancer, angiogenesis inhibitor, IM862, clinical trial

## Abstract

IM862 is a naturally occurring dipeptide (L-glu-L-trp) with immunomodulatory and antiangiogenic properties. A significant anticancer activity has been reported recently in AIDS-related Kaposi's sarcoma, a tumour of endothelial cell origin. The high vascularity and responsiveness to immunotherapy of renal cell carcinoma (RCC) makes such a tumour a potential target for IM862. In all, 25 patients were accrued in a prospective phase II trial using a standard two-step design. The main inclusion criteria were WHO performance status ⩽2, age over 18 years, expected survival >3 months, normal marrow, kidney and liver functions. IM862 was given intranasally at a dose of 20 mg three times daily. Each cycle consisted of 8 consecutive weeks of treatment. All 25 patients were fully evaluable for response and 24 for toxicities. Median age was 62 years (range 42–76), median WHO PS was 1 (0–2). No grade 2 or 3 toxicities related to the study drug have been recorded. Eight patients had stable disease (SD) and 17 progressed while on treatment. Median survival was 7.9 months (range 2.7–20). Median time to progression was 1.9 months (range 1.2–12.6). Median duration of SD was 6 months (range 5.2–12.6+). Analysis of blood angiogenic markers showed a significant decrease of plasma vascular endothelial growth factor (VEGF) levels after 4 and 8 weeks of therapy. Treatment with IM862 has no toxicity, but does not lead to any significant objective responses in metastatic RCC. IM862 should not be further evaluated as a single agent at these doses and schedule for this population of patients. The decrease in VEGF levels warrants further investigation of IM862 as an antiangiogenic therapy.

Renal cell carcinoma (RCC) is resistant to most chemotherapeutic agents (Godley and Ataga, 2000). Although immunotherapy with interleukin-2 (IL-2), interferon *α* (IFN*α*) or both is being considered standard, objective responses are seen only in 10–20% of patients (Bukowski, 1997; Fossa, 2000). Therefore, the identification of new potentially effective agents is of high priority for clinical investigation (Berg *et al*, 2000).

IM862 is a naturally occurring dipeptide (L-glu-L-trp) whose exact mechanisms of action are currently unknown. In animal experiments, dose-dependent increase in natural killer (NK) cells has been observed, together with activation of lymphocytes T4 and T8 and polarisation to the Th1 profile. IM862 has also been shown to be a potent antiangiogenic agent both *in vitro* and *in vivo* (IM862 Investigator's Brochure, 1998; Ferrario *et al*, 2000). A significant anticancer activity has been reported recently in AIDS-related Kaposi's sarcoma, a tumour of endothelial cell origin (Tulpule *et al*, 2000). Responses have also been documented in ovarian carcinoma and skin melanoma (Garcia *et al*, 2001).

Owing to the vascular nature of renal tumours, an antiangiogenic approach is attractive. Furthermore, the responsiveness of RCC to immune therapy makes it a potential target for the antiangiogenic and immunomodulatory agent IM862. We thus initiated a phase II study of IM862 in patients with metastatic RCC using a three times daily nasal administration. We confirm the lack of significant toxicity of this drug. Blood samples were prospectively collected throughout the study to analyse surrogate markers of angiogenesis. We did not observe any objective response, although there were effects on plasma vascular endothelial growth factor (VEGF).

## PATIENTS AND METHODS

### Patients selection

Patients were required to have cytologically and/or histologically proven metastatic RCC, a World Health Organization (WHO) performance status of 0–2, to be over 18 years of age, to have an expected survival of at least 3 months and no history of other invasive cancer. There were no exclusions for prior therapy, but at least 3 weeks had to have elapsed since the last dose of the prior drug or radiotherapy. Patients were also required to have adequate bone marrow, kidney and liver function defined by serum creatinine ⩽170 *μ*mol l^−1^, AST/ALT ⩽3 times the upper limit of normal (ULN), total bilirubin <2 times the ULN, haemoglobin ⩾10 g dl^−1^, WBC ⩾3.10^9^ l^−1^, ANC ⩾2.10^9^ l^−1^ and platelet count ⩾100 × 10^9^ l^−1^. The study was approved by the Central Oxford Research Ethic Committee (COREC) and conducted according to the recommendations of the Declaration of Helsinki and the Association of British Pharmaceutical Industry guidelines for Good Clinical Practice. All patients provided written informed consent prior to study entry. In all, 25 patients were enrolled in the Cancer Research UK Medical Oncology Unit of Oxford.

### Treatment and dose modifications

IM862 was administered intranasally at a dose of 20 mg three times daily throughout the duration of treatment. Each cycle of treatment consisted of 8 weeks of therapy. With the exception of haematological toxicity, any grade 4 toxicity attributable to the study drug led to definitive cessation of treatment. Any grade 3 toxicity led to temporary discontinuation of the study drug and treatment was to be resumed at full dose when toxicity resolved to grade 1. Reoccurrence of the same grade 3 event after rechallenge or unresolved toxicity led to withdrawal from the study.

### Patient follow-up and study end points

The primary end point for this study was objective tumour response rate or sustained disease stabilisation. Response evaluations were performed after an initial 8 weeks of therapy and every 8 weeks thereafter. Earlier evaluations were performed in patients who experienced clinical signs or symptoms suggesting disease progression. Responses were defined according to standard WHO criteria. A complete response (CR) required the complete disappearance of all objective evidence of disease as measured on two separate occasions no less than 4 weeks apart. A partial response (PR) was defined as a more than 50% reduction in the sum of the products of the perpendicular diameters of bidimensional target lesions without any progression of other lesions by more than 25% or appearance of any new lesion. Progressive disease (PD) was defined as the enlargement of any known measurable lesion by more than 25% or the development of any new metastatic lesion. When the criteria for PR or PD were not fulfilled, patients were classified as having stable disease (SD). Toxicity grading was by the NCI common toxicity criteria version 2.0. Toxicity evaluations were performed at 2 and 4 weeks and then every 4 weeks thereafter. Compliance with study medication was documented by interview, returned medication and completion of a patient's diary. Time to progression was calculated from the onset of treatment to the date of first documented disease progression or relapse. Overall survival was calculated from the onset of treatment to the date of death or time to loss of follow-up. Whenever data were censored, estimates were calculated by using the Kaplan–Meier product-limit method. A secondary objective of this study was to assess the role of urine, serum and plasma markers to monitor an antiangiogenic effect.

### Measurement of angiogenic markers

Blood samples were collected prior to the first administration of IM862 and on weeks 2, 4 and 8 and every 8 weeks subsequently. The following markers were assessed: serum and plasma VEGF levels, serum vascular cell adhesion molecule-1 (VCAM-1), E-selectine (E-Sel), von Willebrand's factor antigen (vWF) and inhibitor of urokinase plasminogen activator (PAI-1). The 10-ml blood sample was chilled on ice, centrifuged at 2000 r.p.m. for 10 min at 4°C and stored in 1.5-ml aliquots at −70°C until analysis. Serum VCAM-1, E-selectin and serum/plasma VEGF were measured using ELISA kits supplied by R&D systems (Abingdon, UK). The serum vWF was measured using ELISA kits from American Diagnostica (Greenwich, Connecticut, USA) and a standard vWF from the National Institute for Biological Standards and Controls (Potters Bar, Hertfordshire, UK). Plasma PAI-1 was analysed by ELISA using in-house methods as described previously (Stephens *et al*, 1997). The normal range (or mean±s.d.) for each assay was as published previously: serum VEGF 62–707 pg ml^−1^ (mean 220 pg ml^−1^); plasma VEGF 9–109 pg ml^−1^; plasma PAI-1 8.3–17 U ml^−1^; serum VCAM-1 525±173 ng ml^−1^; serum E-selectin 48.37±19.6 ng ml^−1^; serum vWF 103±30 IU dl^−1^ (Stephens *et al*, 1997; Braybrooke *et al*, 2000). Analysis of the angiogenic markers was made in comparison with a healthy control population without cancer (*n*=39) recruited among asymptomatic hospital workers, age and sex matched for the cases. Exclusion criteria for all control subjects were malignancy, acute or chronic liver and kidney diseases, connective tissue disease, psoriasis, scleroderma, diabetes and symptomatic vascular diseases.

### Statistical considerations

Patient accrual was performed according to a standard two-step design. An initial 14 patients were accrued, with at least one response or alternatively three SD needing to be observed in order to proceed to the second accrual stage with an additional 11 patients. The treatment would be considered worth of further study if two or more of 25 patients experienced an objective response. This design yields a 0.95 or greater probability of accepting the regime if the true response rate is at least 20%, and a 0.69 or greater probability of rejecting the regime if the true response rate is less than 5%. Demographic and baseline characteristics were summarised by descriptive statistics. Efficacy and safety parameters were evaluated in all enrolled subjects. Confidence intervals on response rates were computed using the binomial distribution. The Wilcoxon rank-sum test was used to analyse differences in angiogenic markers before and after 4 and 8 weeks of therapy and Student's *t*-test to compare pretreatment angiogenic marker levels with control values. The analysis was performed using the Stata statistical software, release 5.0 package (Stata Corp., College Station, TX, USA).

## RESULTS

### Patient characteristics

The demographic data, sites of metastatic disease and previous therapies are summarised in Table 1

**Table 1:** Patients characteristics and previous therapies

Number of patients	25
Sex (M/F)	17/8
	
*Age (in years)*	
Median	62
Range	42–76
	
*Performance status (WHO)*	
Median	1
0	9 (36%)
1	11 (44%)
2	5 (20%)
	
*Number of metastatic sites*	
Median	1
1	14
2	9
⩾3	2
	
*Dominant metastatic site*	
Visceral	19 (76%)
Lung only	7 (28%)
Liver (± other sites)	5 (20%)
Other	6 (24%)
Bones (± other sites)	4 (16%)
Lymph nodes only	6 (24%)
	
Prior nephrectomy	15 (60%)
	
*Prior therapy for metastatic disease*	
Immunotherapy	2 (8%)
Antiangiogenic agent	6 (24%)
Medroxyprogesterone acetate (MPA)	4 (16%)
Retinoid (tazarotene)	1 (4%)
Radiotherapy	8 (32%)
	
*Disease-free interval*^a^ *(months)*	
Median	3.7
Range	0–122.2
Nb metastatic at presentation	11
	
*Laboratory studies: median (range)*	
Haemoglobin (g dl^−1^)	12 (9–16)
LDH (IU l^−1^)	173 (110–397; ULN: 250)
Alkaline phosphatase (IU l^−1^)	219 (130–1116; ULN: 250)
Creatinine (*μ*mol l^−1^)	105 (71–188; ULN: 150)

a Time from initial diagnosis to first disease relapse; ULN=upper limit of normal; LDH=lactate dehydrogenase.

. The median age was 62 years (range 42–76) and the median WHO performance status was 1 (range 0–2). Except for six patients, who had lymph nodes metastases only, all patients had visceral metastatic involvement with a median number of metastatic sites of 2 (range 1–4). The median interval from initial diagnosis to first relapse was 3.7 months (range 0–122 months). In all, 11 patients presented with synchronous metastatic disease at the time of initial diagnosis. Two patients had received prior immunotherapy with IFN*α*, six had received prior antiangiogenic agent (razoxane 1, bryostatin 3 and marimastat 2), one had received a retinoid (tazarotene), four had received medroxyprogesterone acetate, 15 had underwent radical nephrectomy. In total, 14 patients were treated in a first-line metastatic setting, nine patients were treated in second line and two in third line. A total of 52 cycles of study treatment were administered to the 25 patients. The median number of treatment cycles was 2 (range 1–6), and the median duration of follow-up at the time of this analysis was 7 months (range 3–20 months).

### Tumour response and survival

**Figure 1:**
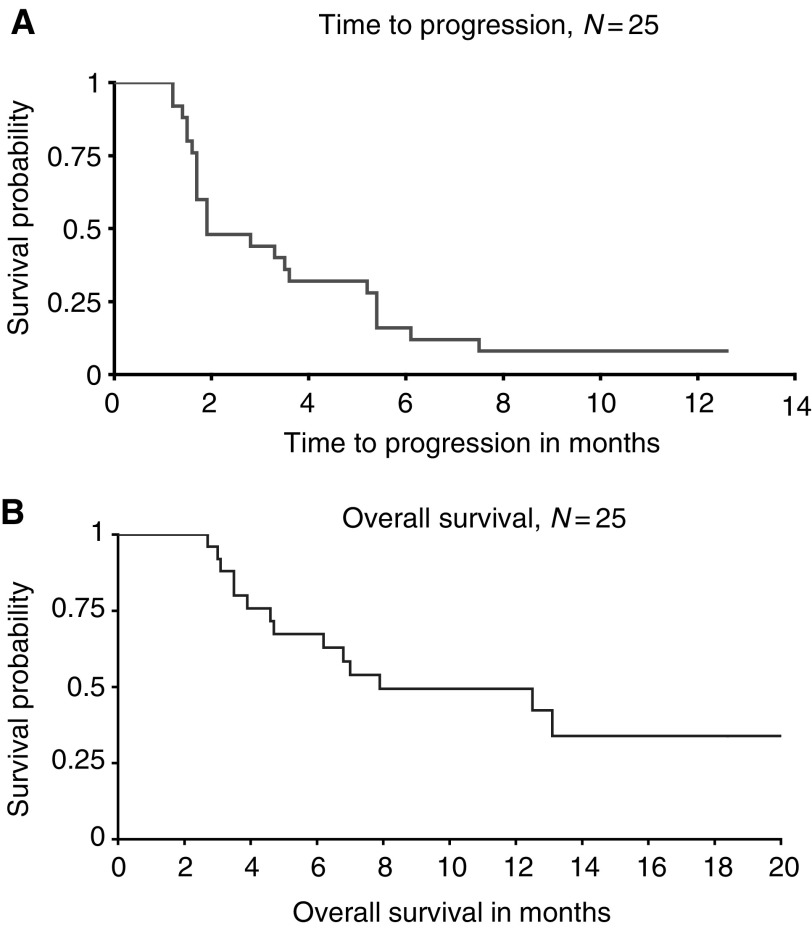
Kaplan–Meier estimates of (**A**) time to progression and (**B**) overall survival for all enrolled patients (*n*=25).

In all, 25 patients were assessable for response. No objective response was observed. Eight patients (32%; 95% CI: 15–53%) had SD, of which three occurred in the first cohort of 14 patients, leading to the extension of the accrual to 25 patients according to the study design. Median duration of SD was 6 months (range 5.2–12.6+). Of note, SDs were observed only in patients with previously documented slowly progressing disease. All patients with synchronous metastatic disease treated in first-line therapy progressed while on the study drug. With a median follow-up duration of 7 months, 14 of all 25 patients have died of disease progression. Only one patient remains on study drug on a compassionate basis at 12.6+ months. The median time to progression was 1.9 months (range 1.2–12.6+ months). The median overall survival was 7.9 months (range 2.7–20 months) (Figure 1

).

### Toxicity

A total of 24 patients, who received a total of 52 cycles (415 weeks of treatment), were assessable for toxicity. One patient included with a baseline performance status of 2 went off study prematurely after 1 week because of a significant deterioration of health, and assessment of toxicity was therefore not available. Side effects associated with treatment are listed in Table 2

**Table 2:** Worst toxicity grade per patient (*n*=24) across all 52 cycles

	**Toxicity grade**				
**Toxicity**	**0**	**1**	**2**	**3**	**4**
Fatigue/asthenia	4	9	6	5	—
Headache	20	4	—	—	—
Pain	13	7	3	1	—
Anorexia	23	1	—	—	—
Nausea	13	4	5	2	—
Vomiting	16	4	1	3	—
Sinusitis	22	1	1	—	—

. Although several grade 3 toxicities were reported, none were related to the study drug in the investigators' opinion. The only concern was for a patient with known ulcerative colitis, who experienced a flare of his digestive symptoms while on treatment. The drug was discontinued and symptoms resolved, but after treatment with IM862 was resumed his symptoms exacerbated again, leading to definitive cessation of the drug and the patient went off study. Relationship of this event to the study drug could not be ascertained, but was felt to be possibly related. Overall, treatment was discontinued prematurely because of toxicity concern for this patient only. For all other cases, no treatment delay nor dose reduction were necessary. Toxicities probably related to the study drug included two episodes of, respectively, grade 1 and 2 sinusitis and four patients experiencing grade 1 headache.

### Analysis of angiogenic markers

The results for the plasma/serum surrogate markers of angiogenesis are summarised in Table 3

**Table 3:** Summary of changes in plasma/serum markers of angiogenesis

	**Baseline**			
**Marker**	**Controls (*n*=39)**	**Patients (*n*=23)**	**Week 4**	**Week 8**
Serum VEGF (pg ml^−1^)	20 (1199.3)	185 (218.552)*	170 (60.600)	180 (213.326)
Plasma VEGF (pg ml^−1^)	70 (221.024)	280 (555.927)*	200 (486.197)**	120 (177.708)**
Serum VCAM-1 (ng ml^−1^)	460 (133.83)	640 (165.6)*	620 (182.6)	645 (256.3)
Serum E-selectin (ng ml^−1^)	42 (18.37)	43.5 (19.6)	43 (19.2)	44 (25.2)
Serum vWF (IU dl^−1^)	103 (21.82)	138 (26.9)*	138 (29.6)	146 (27.9)
Plasma PAI-1 (*μ*g l^−1^)	7.1 (5.1)	16.5 (10.5)*	15 (12.7)	14 (11.7)

Median values (standard deviation).

* Significantly increased (*P*<0.05).

** Significant decrease (*P*<0.05).

. Blood samples from only 23 patients were fully available at week 8 to allow for meaningful analysis and comparison between baseline and controls at 4 or 8 weeks of therapy. As compared to a healthy control group, we found at baseline, prior to therapy, significantly elevated levels of serum and plasma VEGF, serum VCAM-1 and vWF and plasma PAI-1 for renal cell cancer patients (serum VEGF, *P*=0.0004, rank-sum test; plasma VEGF, *P*=0.0003, Mann–Whitney test; serum VCAM-1, *P*=0.0082, Student's *t*-test; serum vWF, *P*=0.0018, Student's *t*-test; plasma PAI-1, *P*=0.0040, Student's *t*-test). After 4 and 8 weeks on therapy, the only effect was seen on the levels of plasma VEGF, with a significant decrease from 280 pg ml^−1^ prior to therapy to 200 pg ml^−1^ at week 4 (*P*=0.02, signrank test) and 120 pg ml^−1^ at week 8 (*P*=0.0065, signrank test). Therefore, the levels of plasma VEGF seemed to decrease with duration of treatment. Whatever the angiogenic marker considered, there was no significant difference between stable and progressing patients in the levels of any marker, whether at baseline or after 4 or 8 weeks on therapy (Table 4

**Table 4:** Comparison of plasma/serum markers of angiogenesis between patients with stable or progressive disease

	**Baseline (*n*=23)**		**Week 8 (*n*=23)**		***P***	
**Marker**	**Stable disease**	**Progressive disease**	**Stable disease**	**Progressive disease**	**Baseline**	**8 weeks**
Serum VEGF (pg ml^−1^)	200 (100.162)	180 (276.462)	165 (86.848)	180 (276.458)	NS*	NS*
Plasma VEGF (pg ml^−1^)	300 (550.492)	210 (578.123)	210 (409.944)	100 (575.927)	NS*	NS*
Serum VCAM-1 (ng ml^−1^)	660 (151)	600 (175)	650 (263)	610 (258)	NS**	NS**
Serum E-selectin (ng ml^−1^)	42 (14)	46 (23)	46 (29)	44 (23)	NS**	NS**
Serum vWF (IU dl^−1^)	128 (34.6)	144.5 (19.8)	148.5 (32.8)	146 (25)	NS**	NS*
Plasma uPAsr (*μ*g l^−1^)	20 (11)	14.3 (9.7)	15.6 (13.8)	13 (9.4)	NS**	NS**

Median values (standard deviation).

* Values calculated by ranksum test.

** Values calculated by Student's *t*-test. NS=not significant (*P*>0.05).

). For example, the decrease in plasma VEGF levels between baseline and week 8 was, respectively, 300 *vs* 210 for SD and 210 *vs* 100 pg ml^−1^ for PD.

## DISCUSSION

Since the first reports in the mid-80s of effective immunotherapy with IFN*α* and subsequently IL-2 for metastatic RCC, little progress has been really made and response rates are still plateauing around 15% in large multicentre trials (Hernberg *et al*, 1999). There is therefore an urgent need for identification of new effective agents and especially those representing novel paradigms such as targeting of tumour angiogenesis. The rationale for using IM862 was its known immunomodulatory and antiangiogenic properties, making this compound a good candidate for treatment of a highly vascularised tumour responsive to immunotherapy. Furthermore, a demonstrated activity of IM862 in AIDS-related Kaposi's sarcoma (Tulpule *et al*, 2000) together with minimal toxicity, especially with regard to the many side effects encountered with IL-2 and IFN*α*, prompted us to initiate this phase II trial.

We were able to confirm, like others (Tulpule *et al*, 2000; Garcia *et al*, 2001), the lack of significant toxicity of IM862. We did not observe any objective response. Moreover, the design of our trial allowed for SD (no change for at least 8 weeks) to account as a clinical benefit, and the observation of three SDs at 8 weeks in the first part of the study led to extension of accrual to 25 patients. Despite this, a careful review of definitive results showed that SD had been observed only in pretreated patients and not in patients with synchronous metastatic disease. This could mean that the recorded SD might only reflect the natural history of a slowly progressing disease.

Many hypotheses could account for a lack of effectiveness of IM862 towards RCC in our study. First, the dosing and schedule used could be inadequate and we had no way to evaluate if therapeutically active concentrations of IM862 had been reached, such as by measuring IM862 blood levels or modulation of Th1 lymphocyte subset. However, Tulpule *et al* (2000) used a lower dose of 5 mg given intranasally once daily every other day or for 5 days, followed by 5 days of rest. For this lower concentration, a demonstrable clinical effect was recorded in terms of response rate. The optimal dose for treatment of solid tumour remains to be determined. Second, RCC could represent a naturally resistant tumour to antiangiogenic treatment. This seems unlikely, as in theory tumour blood vessels are genetically stable and therefore should not exhibit any resistance to an effective antiangiogenic therapy. In addition, several other antiangiogenic compounds such as razoxane, bryostatin-1, TNP-470, or anti-VEGF antibodies have shown some activity with even objective tumour responses in RCC (Stadler *et al*, 1999; Braybrooke *et al*, 2000; Pagliaro *et al*, 2000; Yang *et al*, 2003). Third, the primary end point, objective response rate, could be inadequate to assess the efficacy of an antiangiogenic agent which is in theory thought to be more cytostatic than cytotoxic. A surrogate end point could have been, for example, time to progression, such as in the pivotal randomised phase II study recently reported by Yang *et al* (2003), which showed some benefit of bevacizumab in metastatic RCC. However, at the time when this study was originally designed, this was not a commonly accepted end point. Moreover, in this randomised phase II study, only the high-dose bevacizumab arm showed a significant effect over placebo and was associated with a 10% ORR. Similarly, the effectiveness of bevacizumab in combination with chemotherapy for metastatic colon cancer was seen with a better response rate, time to progression and overall survival as compared to chemotherapy alone (Hurwitz *et al*, 2004). Therefore, to achieve an objective response with an antiangiogenic compound does not seem unrealistic, and the fact that Tulpule *et al* (2000) reported objective responses in Kaposi's sarcoma with IM862 initially prompted us to choose response rate as the primary objective in our study. But, as already mentioned earlier, we allowed for SD to account as a clinical benefit in the design of our trial, thus anticipating a potential effect other than tumour shrinkage.

The median time to progression and overall survival of, respectively, 1.9 and 7.9 months are similar to those reported earlier in the same institution for a larger phase II study involving another antiangiogenic drug (Braybrooke *et al*, 2000). Moreover, despite the lack of effectiveness in term of response rate, the observed median overall survival is similar to the one reported in the MRC phase III randomised trial, comparing IFN*α* (8.5 months) to medroxyprogesterone acetate, with obviously a toxicity profile that would favour IM862 (MRC Renal Cancer Collaborators, 1999). Therefore, even if no responses were observed, one cannot completely rule out some activity of IM862 in term of prolongation of SD or time to progression, as it has been recently reported for bevacizumab (Yang *et al*, 2003). The answer to such a question would only be clarified in a larger randomised, placebo-controlled trial.

The evaluation of serum and plasma markers of angiogenesis may have a role to play in assessing the efficacy of novel antiangiogenic drugs (Harris, 1997; Deplanque and Harris, 2000). Elevated levels of serum VEGF, E-selectin and VCAM-1, and of plasma PAI-1 and VEGF, have already been linked to a worse prognosis in a variety of cancers (Kuroi and Toi, 2001). In a previous study, we were able to find a significant difference in the levels of serum VCAM-1 between patients with PD compared to those with SD (Braybrooke *et al*, 2000). This was not the case in the present study, maybe due to the smaller number of patients or the different antiangiogenic agent used. Similarly, we could not find any difference in the levels of the other angiogenic markers between patients with PD and SD. However, a significant decline of plasma VEGF levels was observed after 4 and 8 weeks of therapy for all patients as compared to pretreatment levels. This significant decline in plasma VEGF levels would make IM862 an antiangiogenic agent worth of consideration for further investigation. Effectively, some preliminary studies have linked VEGF levels with tumour burden in colon carcinoma (Nakayama *et al*, 2002; De Vita *et al*, 2004). Interestingly, VEGF levels dropped significantly after surgery and persistent elevated VEGF levels correlated with relapse, indicating significant residual disease. If lowering of VEGF levels by IM862 could be further confirmed in other studies, this could form the basis for designing clinical trials in other settings such as minimal residual disease after tumour debulking, that is, in an adjuvant setting. Moreover, the apparent lack of significant toxicity of IM862 would make it an attractive candidate for combination with other chemotherapy or biologic agents. Indeed, combination of an antiangiogenic agent with low-dose chemotherapy is highly synergistic in preclinical models (Klement *et al*, 2000).

In conclusion, IM862 has no activity as a single agent against RCC, at least with the dosing and schedule used in our study and if response rate is considered a primary end point. However, because of its lack of toxicity and preliminary data showing some activity in other types of cancer, combination of IM862 with other drugs such as chemotherapy or biologic agents could be worth considering, especially if a biological effect such as the decrease of VEGF levels could be confirmed.
